# Neuroimaging in Atypical AD: Considerations for Clinical Trials

**DOI:** 10.1002/alz70856_098250

**Published:** 2025-12-24

**Authors:** Jennifer L. Whitwell

**Affiliations:** ^1^ Mayo Clinic, Rochester, MN, USA

## Abstract

Neuroimaging can assess different pathophysiological features of the brain in Alzheimer's disease (AD), including structure, function and protein deposition, and can be used to help identify patients for clinical treatment trials and monitor and assess potential treatment effects on disease progression. While large scale longitudinal studies are underway to validate potential neuroimaging biomarkers in amnestic AD, less efforts have focused on atypical clinical presentations, such as language, visual, dysexecutive, behavioral and motor presentations of AD. These patients would be ideal candidates for treatment trials given that they are relatively young, and the disease is less influenced by age‐associated co‐pathologies, providing a purer disease model of AD. Rates of brain atrophy provide excellent surrogate disease markers in amnestic AD and they will also likely have utility in atypical AD, although cortical, rather than medial temporal, measurements perform better. Optimum sample size estimates required to detect a 20% treatment effect in both the language and visual variants of AD were obtained with rates of inferior temporal atrophy in our sample (113 and 100 patients required per arm, respectively). However, heterogeneity in rates and patterns of atrophy are observed across phenotypes, and with age and disease stage, and these factors would need to be considered in clinical trials. Utilizing direct markers of tau deposition from PET would be optimal. Tau uptake increases over time in relatively consistent patterns across atypical AD phenotypes, although rates vary across patients and with age and baseline levels, with tau uptake plateauing at high levels potentially limiting utility as an outcome measure in patients with advanced disease. Metrics of connectivity also worsen over time in atypical AD patients, with evidence that declines in default mode network connectivity may be common across phenotypes and have some utility, although once again changes are highly variable and influenced by age and tau burden. In summary, we are gaining a better understanding of factors that influence rates of change in atypical AD that need to be considered in treatment trials, although more work is needed to identify optimum biomarkers to track disease progression across the AD clinical spectrum.

